# British Schools

**Published:** 1895-11

**Authors:** 


### BRITISH VETERINARY SCHOOLS.

THE London and the two Edinburgh schools were opened for the current collegiate year under favorable aspects. The students at London were addressed by Prof. Penberthy; those of the Dick Veterinary College by the new principnl, Prof. Dewar, and at the New Veterinary College by Richard Rutherford, F.R.C.S. They all spoke of the future of the profession and the rapid changes of the past, the aspect of the public and the great need of prompt and thorough action in the control of bovine tuberculosis, and greater need of safeguards in the meat and milk supply. The English Government officials rightfully received a thorough round of critlcism for their ignoring of the veterinary profession in the Animals Department of the Board of Agriculture, all of which was richly deserved. Professors Dewar and Rutherford pointed to the value of tuberculin as a diagnostic agent, and urged its use by the Department of Health, to enable a weeding-out of diseased animals.

